# Retraction: Carbon enters silica forming a cristobalite-type CO_2_–SiO_2_ solid solution

**DOI:** 10.1038/ncomms13417

**Published:** 2016-11-29

**Authors:** Mario Santoro, Federico A. Gorelli, Roberto Bini, Ashkan Salamat, Gaston Garbarino, Claire Levelut, Olivier Cambon, Julien Haines

Nature Communications 5: Article number: 3761; DOI: 10.1038/ncomms4761 (2014); Published: 04 30 2014; Updated: 11 29 2016

In this Article, we reported the synthesis of a crystalline CO_2_–SiO_2_ solid solution by reacting carbon dioxide and silica in a laser-heated diamond anvil cell at pressures between 16 and 22 GPa and temperatures greater than 4,000 K, and showed that carbon enters silica. We have now reanalysed all our X-ray diffraction patterns, in particular those at room pressure where potential volatile components (for example, CO_2_) are absent, making data interpretation as simple and clean as possible^1^. Indeed, we find that orthorhombic β-ReO_2_ (Pbcn) provides a better fit to the temperature-quenched new phase than tetragonal cristobalite. This possibility was also indicated by experimental results from Santamaria-Perez and co-workers^2^. Hence what we previously interpreted as a CO_2_–SiO_2_ solid solution now appears to be ReO_2_, indicating the decomposition of CO_2_ leading to the oxidation of Re from the gasket, which is found to diffuse into the sample in the laser heating experiment. The authors therefore wish to retract this Article.
